# Feasibility of quantifying *SDC2* methylation in stool DNA for early detection of colorectal cancer

**DOI:** 10.1186/s13148-017-0426-3

**Published:** 2017-12-04

**Authors:** Tae Jeong Oh, Hyun Il Oh, Yang Yei Seo, Dongjun Jeong, Changjin Kim, Hyoun Woo Kang, Yoon Dae Han, Hyun Cheol Chung, Nam Kyu Kim, Sungwhan An

**Affiliations:** 1 Genomictree, Inc, 44-6 Techno 10-ro Yuseong-gu, Daejeon, 34027 South Korea; 2 Department of Pathology, College of Medicine, Soonchunhyang University, 23-20 Byeongmyeong-dong Dongnam-gu, Cheonan, Chungcheongnam-do 31151 South Korea; 3 Department of Internal Medicine, Dongguk University Ilsan Hospital, College of Medicine, Dongguk University, 27 Dongguk-ro Ilsandong-gu, Goyang-si, Gyeonggi-do 10326 South Korea; 4 Department of Surgery, Yonsei University College of Medicine, 50-1 Yonsei-ro Seodaemun-gu, Seoul, 03722 South Korea; 5 Yonsei Cancer Center Yonsei University College of Medicine, 50-1 Yonsei-ro Seodaemun-gu, Seoul, 03722 South Korea

**Keywords:** Colorectal cancer, Early detection, Methylation, Precancerous lesion, *SDC2*, Stool DNA

## Abstract

**Background:**

Colorectal cancer (CRC) screening is the most efficient strategy to reduce disease-related mortality. Frequent aberrant DNA methylation is known to occur in selected genes and early during CRC development, which has emerged as a new epigenetic biomarker for early detection of CRC. Previously, we reported that we identified that CpG sites of *SDC2* were aberrantly methylated in tumor tissues of most CRC patients through comprehensive methylation analysis and demonstrated a high potential of quantification of *SDC2* methylation in blood for early detection of colorectal cancer. In this study, we aim to investigate the feasibility of quantifying *SDC2* methylation in stool DNA for the early detection of CRC. The objective of this study was to confirm a high frequency of *SDC2* methylation in tumor tissues at various stages of CRC and investigate the feasibility of a quantitative test for *SDC2* methylation in fecal DNA by highly sensitive and accurate real-time PCR for early detection of CRC.

**Methods:**

Bisulfite-pyrosequencing assay was performed to measure the *SDC2* methylation status in tissue samples. For methylation analysis in stool DNA, a highly sensitive and accurate method was applied which implements consecutive two rounds of PCR consisting of unidirectional linear target enrichment (LTE) of *SDC2* and quantitative methylation-specific real time PCR (qMSP) for *SDC2*, named as me*SDC2* LTE-qMSP assay. Its limit of detection was 0.1% methylation (corresponding to ~ 6 copies in total ~ 6200 genome copies).

**Results:**

Positive *SDC2* methylation was observed in 100% of primary tumors, 90.6% of adenomatous polyps, 94.1% of hyperplastic polyps, and 0% of normal tissues. *SDC2* methylation level also significantly (*P* < 0.01) increased according to the severity of lesions. In stool DNA test for *SDC2* methylation by LTE-qMSP comparing CRC patients with various stages (I to IV) (*n* = 50) and precancerous lesions (*n* = 21) with healthy subjects (*n* = 22), the overall sensitivity was 90.0% for detecting CRC and 33.3% for detecting small polyps, with a specificity of 90.9%.

**Conclusions:**

Taken together, our result indicates that stool DNA-based *SDC2* methylation test by LTE-qMSP is a potential noninvasive diagnostic tool for early detection of CRC.

## Background

Colorectal cancer (CRC) is the second most common cause of malignant deaths in industrialized countries, and it is known to be major cause of cancer morbidity and mortality [1, 2]. The 5-year survival rate for CRC can be as high as 90%, if the cancer is detected at an early stage, but it is estimated to be less than 10% if a metastasis occurs [3]. Several CRC screening tools have been developed to facilitate the early detection of CRC, including colonoscopy and fecal occult blood testing (FOBT) [4]. For the last 10 years, colonoscopy has been preferred as a screening tool to detect CRC early [5]. However, the acceptance of screening colonoscopy remains very low within the general public, for several reasons including the need for extensive bowel preparation [4]. Meanwhile, noninvasive stool tests such as immunochemical FOBT have been available so far as an attractive alternative to screen people for colonoscopy referral. However, this has limited use as a screening test to detect of earlier stages of CRC because of its low sensitivity in detecting stage I of CRC and advanced adenoma, at 53 and 27%, respectively [6]. Thus, significant efforts have been made to develop noninvasive molecular tests using accurate molecular biomarkers to detect CRC and colorectal adenomas at an early stage [7].

The aberrant methylation of genes is an epigenetic change that induces gene silencing of tumor suppressor genes, and it has been recognized as one of the most common molecular alterations in CRC and in other human cancers [8–10]. Methylated DNA is known to be chemically and biologically stable, it is less subjected to transient alterations, and it is readily detectable in many types of body fluids including blood and stool. Therefore, aberrantly methylated specific DNA sites in solid tumors are well suited as noninvasive molecular diagnostics for the early cancer detection [11–13].

Several stool-based DNA methylation markers such as *TFPI2*, *VIM*, *SFRP2*, *NDRG4*, *BMP3*, and *SDC2* have been previously described as potential markers for early CRC detection [14–18]. Overall, these reports presented sensitivities of 46 to 89% and specificities of 76.8 to 93%. Notably Imperiale et al. [19] recently reported a new stool DNA test to measure two methylation biomarkers and seven site mutations of *KRAS* in addition to a hemoglobin test in the stool sample. This combinatorial test showed an overall sensitivity of 92% with a specificity of 87% for CRC detection, and it was approved by the US FDA in 2014.

We previously determined that normally unmethylated CpG sites of *SDC2* are predominantly methylated in tumor tissues of CRC and subsequently demonstrated that the aberrant methylation of *SDC2* is frequently detected in serum DNA derived from CRC patients, but rarely in healthy subjects, indicating potential as a biomarker for early diagnosis of CRC [20]. The syndecan-2 (*SDC2*) protein functions as an integral membrane protein and participates in cell proliferation, cell migration, and cell-matrix interactions via its receptor for extracellular matrix proteins [21, 22]. In this study, we used a bisulfite-pyrosequencing methylation assay on an independent group of CRC patients to confirm the high prevalence of aberrant *SDC2* methylation in tumor tissues of CRC patients and precancerous biopsies with various stages compared to those of normal tissues. For the clinical validity of stool-based *SDC2* methylation assay in detecting CRC, we introduced a very sensitive and accurate method that consists of quantitative methylation-specific PCR coupled with linear target enrichment (LTE-qMSP). The clinical validity of the early CRC detection using LTE-qMSP for *SDC2* methylation in stool DNA was assessed by comparing observation for patients with various stages of CRC and precancerous lesions to those of healthy individuals. The results indicated that *SDC2* methylation has high potential as a biomarker useful in noninvasive diagnostics of early-stage CRC.

## Methods

### Reagents

All chemical reagents used were purchased from Sigma-Aldrich (MO, USA) unless otherwise noted. Oligonucleotides and fluorescent probes were synthesized by Integrated DNA Technologies (Iowa, USA).

### Cell line and clinical specimens

Human colon cancer cell HCT116, SW480, and HT-29 were obtained from Korean Cell Line Bank (Seoul, South Korea) and maintained in RPMI 1640 medium (JBI, Seoul, South Korea) supplemented with 10% fetal bovine serum (JBI, Seoul, South Korea), 100 unit/mL of penicillin (JBI, Seoul, South Korea), and 100 μg/mL of streptomycin (JBI, Seoul, South Korea) in a humidified incubator at 37 °C with 5% CO_2_.

Paraffin sections of polyp tissues (*n* = 49) and fresh-frozen sections of primary colorectal tumor tissues (*n* = 18) were obtained from College of Medicine, Soonchunhyang University (Chonan, South Korea). Briefly, tissue blocks were cut with a standard microtome (Leica Biosystems, Nussloch, Germany) to generate successive sections. They were mounted onto adhesive silane-coated slides. Polyp and tumor components were identified and marked on hematoxylin and eosin stained sections for further DNA studies by an expert surgical pathologist. Genomic DNA (*n* = 5) from normal mucosa without any history of malignancy were purchased from BioChain Institute (CA, USA).

Stool samples from patients with histology-confirmed CRC (*n* = 50), adenomatous polyps (*n* = 21), and healthy normal subjects without any history of malignancy (*n* = 22) were obtained from Cancer Center of Yonsei University College of Medicine (Seoul, South Korea) and Dongguk University Ilsan Hospital (Ilsan, South Korea). All stool samples were collected prior to colonoscopy or purgative bowel preparation. The collection paper (JeongHyun MED, Seoul, South Korea) mounted to the toilet seat was used to prevent contamination of toilet water. Approximately 10 g of a single stool from each individual was collected from four to five different spots in 20 mL of preservative buffer (Genomictree, Inc., Daejeon, South Korea) using spatula. All stool samples were stored at 4 °C until used. Detailed characteristics of enrolled patients are summarized in Table 1. This study was approved by the Institutional Review Board of College of Medicine of Soonchunhyang University, Dongguk University Ilsan Hospital, and Cancer Center of Yonsei University College of Medicine. Written informed consent was obtained from all study participants, adhering to local ethics guidelines.

**Table 1:** Clinicopathological features of colorectal tissue and stool samples used in this study

Characteristics	Number of patients (%)	
	Tissues	Stool
Healthy normal	*n* = 5	*n =* 22
Sex (%)		
Male	4 (80.0)	12 (54.5)
Female	1 (20.0)	10 (45.5)
Age, mean (range)	55.5 (48–65)	58.8 (36–77)
Polyp	*n* = 49	*n* = 21
Sex (%)		
Male	37 (75.5)	13 (61.9)
Female	12 (24.5)	8 (38.1)
Age, mean (range)	57.2 (28–89)	63.4 (51–75)
Number of polyps analyzed from each patient (range, 1–6) (%)		
1	21 (42.9)	3 (14.3)
2–3	23 (46.9)	15 (71.4)
≥ 4	5 (10.2)	3 (14.3)
Histopathology		
Hyperplastic	17 (34.7)	
Tubular	27 (84.3)	21 (100)
Tubularvillous	5 (15.7)	
CRC	*n* = 18	*n* = 50
Sex (%)		
Male	8 (40.0)	30 (60.0)
Female	12 (60.0)	20 (40.0)
Age, mean (range)	63.1 (39–80)	61.9 (41–84)
Stage (%)		
I	2 (10.0)	12 (24.0)
II	12 (60.0)	17 (34.0)
III	2 (10.0)	10 (20.0)
IV	4 (20.0)	11 (22.0)

### DNA isolation

For fresh-frozen tissues, specimens were embedded in Tissue-Tek O.C.T compound (Sakura Finetek, Torrance, USA) and frozen at − 20 °C in cryostat chamber (Leica Biosystems, Nussloch, Germany). Five to seven frozen tissue sections (12 μm in thickness) and four to six sections (10 μm in thickness) of formalin-fixed paraffin-embedded (FFPE) tissues were prepared using microtome (Leica Biosystems, Nussloch, Germany). To remove paraffin for FFPE sections, 1.0 mL of xylene was added to paraffin sections in 2.0 mL of microcentrifuge tubes and incubated at room temperature for 10 min. Tubes were centrifuged at 12,000×*g* for 10 min at room temperature, and the supernatant was discarded. This process was repeated until paraffin was fully removed. One milliliter of ethanol was added to each tube and vigorously shaken to wash out xylene, followed by centrifugation at 12,000×*g* for 10 min. This was repeated three times. All genomic DNA was isolated from tissues and cell lines using QiaAmp DNA Mini kit (QIAGEN, Hilden, Germany) according to manufacturer’s instructions.

All stool samples were washed in excess volume of PBS at once and homogenized in EDTA-based buffer solution (1 mL solution per 0.6 g of stool) with a shaker device. After homogenization, 1 g equivalent of each stool was centrifuged. The supernatants were removed and added 1.0 to 1.4 mL of lysis buffer 1 (Genomictree, Inc., Daejeon, South Korea) to the pellet and incubated for 4 min at room temperature. The samples were centrifuged and removed the supernatant, followed by addition of lysis buffer 2 (Genomictree, Inc., Daejeon, South Korea). The samples were incubated for 2 min at room temperature and centrifuged. 0.75 mL of the supernatant was aliquoted and added 25 μL of proteinase K (0.4 mg/mL) and incubated at 70 °C for 10 min. The samples were subsequently extracted with tris-saturated phenol-chloroform-isoamylalcohol (25:24:1 by volume) (Thermo Scientific, MA, USA). Total nucleic acids were then precipitated (1/10 volume of 3 mol/L sodium acetate and equal volume of isopropanol, removed from solution by centrifugation. The DNA pellet was washed with 70% ethanol and dried. The DNA samples were dissolved in 50 to 100 μL of TE buffer and stored at − 20 °C. Stool DNA was also extracted using QIAamp DNA Stool Mini Kit (QIAGEN, Hilden, Germany) according to manufacturer’s instruction and finally eluted with 50 μL of elution buffer. Initial homogenization steps are same and 0.2 g of stool was used for DNA extraction. The DNA concentration was measured by Qubit dsDNA BR assay kit (Thermo Scientific, MA, USA).

### Bisulfite treatment

Genomic DNA was chemically modified with sodium bisulfite to convert all unmethylated cytosine to uracil while leaving methylated cytosine unmodified using EZ DNA Methylation-Gold kit (ZYMO Research, CA, USA) according to manufacturer’s instructions. Briefly, genomic DNA was treated with sodium bisulfite at 65 °C for 2.5 h. Desulfonation was performed at room temperature for 20 min. Bisulfite-converted DNA was purified using Zymo-Spin IC column (Zymo Research, CA, USA) and eluted with 10 μL of distilled water. The eluted DNA was either used immediately for methylation analysis or stored at − 20 °C until further use.

### Bisulfite-pyrosequencing methylation assay of *SDC2* gene in tissues

To quantify methylation levels of *SDC2*, quantitative bisulfite-pyrosequencing was performed. Specific bisulfite PCR and pyrosequencing primers were designed to analyze 149 bp of 5′ regulatory region including four CpG dinucleotides sites (+456, +460, +466, +473 bp) of *SDC2* gene using PyroMark Assay Design Software v. 2.0 (QIAGEN, Hilden, Germany). The following primers were used: forward, 5′- GGGAGTAGGAGTAGGAGGAGGAA-3′; reverse, 5′- Biotin-ACCAAAACAAAA CCAAACCTCCTACCCA-3′; sequencing primer, 5′-AGGAGGAGGAAGAGAG-3′. Briefly, 20 ng of bisulfite-modified DNA was amplified in a 25 μL reaction volume with gene-specific primers using PyroMark PCR kit (QIAGEN, Hilden, Germany). Samples were heated to 94°C for 10 min and then amplified for 45 cycles at 94°C for 30 s, 56°C for 45 s, and 72°C for 40 s. All reactions were then incubated at 72°C for 10 min for final extension. Pyrosequencing was performed using PyroMark Gold Q96 reagent and PyroMark ID96 instrument (QIAGEN, Hilden, Germany) following manufacturer’s instructions. Briefly, 25 μL of each biotinylated PCR product was immobilized on streptavidin-coated Sepharose HP beads (Amersham Biosciences, NJ, USA) and then subjected to sequencing using automatically generated nucleotide dispensation order of “sequence to analyze” corresponding to each reaction.

Each CpG site was assigned a percentage (%) of methylation by evaluating C/T ratio as methylation index (MtI). The average % of methylation across four CpG sites was obtained. Methylated non-CpG cytosines were used as internal controls to check the fidelity of bisulfite conversion. If MtI of each sample was greater than 5% of detection limit of pyrosequencing [23], it was considered as methylation-positive.

### Analytic performance of me*SDC2* LTE-qMSP

Analytic performance of me*SDC2* LTE-qMSP was determined for PCR replicate by testing aliquots of DNA. To examine the limit of detection (LoD) of *SDC2*-methylated DNA, HCT116 genomic DNA as fully methylated human genomic DNA was serially diluted with an unmethylated genomic DNA. The unmethylated genomic DNA was prepared by whole genome amplification of human lymphocyte genomic DNA (BioChain Institute Inc., CA, USA) using Illustra GenomiPhi V2 DNA Amplification kit (GE Healthcare, OH, USA) [24]. For comparison of me*SDC2* LTE-qMSP with our previous me*SDC2*-qMSP assay [20], different amounts (200, 100, 50, 20, 10, and 0 pg) of fully methylated HCT116 genomic DNA were diluted into unmethylated genomic DNA in total 20 ng of genomic DNA to create mixtures with methylation percentages of 1.0, 0.5, 0.25, 0.1, 0.05, and 0%. Resultant DNA samples from each concentration were pooled and divided to multiple aliquots so that the same DNA substrate was used in PCR for comparison. me*SDC2*-qMSP and me*SDC2* LTE-qMSP were performed in 24 replicates (8 replicates in 3 independent runs using the same real-time PCR instrument on the same day). Primers and probes used for me*SDC2* LTE-qMSP and me*SDC2*-qMSP are listed in Table 2.

**Table 2:** Comparison of me*SDC2* LTE-qMSP with me*SDC2*-qMSP

Description	me*SDC2* LTE-qMSP	me*SDC2*-qMSP
*SDC2* Primer sequences^a^	5′-G**T** **A** **G** **A** **A** **A** **T** **T** **A** **A** **T** **A** **A** **G** **T** **G** **A** **G** **A** **G** **G** **G** **C**-3′ 5′-*AAAGATTCGGCGACCACCGA*A**CG** **A** **C** **T** **C** **A** **A** **A** **C** **T** **CG** **A** **A** **A** **A** **C** **T** **CG**-3′	F: 5′-TAGAAATTAATAAGTGAGAGGGCGT-3′ R: 5′-GACTCAAACTCGAAAACTCGAA-3′
*SDC2* probe^a^	5′-FAM-TTCGGGGCGTAGTTGCGGGCGG-3′	5′-FAM-AGTAGGCGTAGGAGGAGGAAGCGA-3′
*SDC2* amplicon size	124 bp	121 bp
Real time PCR reagents	AptaTaq DNA Master, 5X	Rotor-Gene Probe PCR Kit
Thermal cycling condition	LTE: 95 °C 5 min; 95 °C 15 s, 60 °C 1 min, 35 cycles; heating and cooling rates were 15 and 20 °C/s. qMSP: 95 °C 5 min; 95 °C 15 s, 60 °C 1 min, 40 cycles; heating and cooling rates were 15 and 20 °C/s.	qMSP: 95 °C 5 min; 95 °C 10 s, 62 °C 15 s, 72 °C 20 s; 40 cycles; heating and cooling rates were 15 and 20 °C/s.
Control gene	*COL2A1*	*ACTB*
Primer sequences^a^	5′-GTAATGTTAGGAGTATTTTGTGGITA-3′ 5′-*AAAGATTCGGCGACCACCGA*CTAICCCAAA AAAACCCAATCCTA-3′	F: 5′-TGGTGATGGAGGAGGTTTAGTAAGT-3′ R: 5′-AACCAATAAAACCTACTCCTCCCTT AA-3′
*COL2A1* probe^a^	5′-Cy5-AGAAGAAGGGAGGGGTGTTAGGAGAGG-3′	5′-TET-ACCACCACCCAACACACAATAACAAACA CA-3′
*COL2A1* amplicon size	86 bp	133 bp

*Bold* identical nucleotide sequences with me*SDC2*-qMSP assay, *italics* universal sequence, *I* inosine nucleotide, *F* and *R* indicate forward and reverse primers, respectively

^a^CpG dinucleotide sites are underlined

### Methylation measurement in stool DNA by me*SDC2* LTE-qMSP

For measurement of *SDC2* methylation, LTE was introduced in step 1 in order to specifically enrich methylated *SDC2* target DNA from bisulfite-modified DNA. Additionally, the region lacking CpG dinucleotides of *COL2A1* gene was used as a control [25] for estimation of the amount of amplifiable template and adequacy of bisulfite conversion. A total of 20 μL of reaction mixture contained 2.0 μg of bisulfite-converted stool DNA, each 0.05 μM of *SDC2* methylation-specific antisense and *COL2A1* gene-specific antisense primers attached to 5′ universal sequence, and 4 μL of 5× AptaTaq PCR master mix (Roche Diagnostics, Basel, Swiss). Thermal cycling conditions were as follows: 95 °C for 5 min followed by 35 cycles of 95 °C for 15 s and 60 °C for 60 s. After LTE, the reaction mixture volume was scaled up to 40 μL, containing 8 μL of 5× AptaTaq PCR master mix, 0.25 μM of *SDC2* methylation-specific sense primer, 0.125 μM of *SDC2* probe (FAM), 0.125 μM of *COL2A1* sense primer, 62.5 nM of *COL2A1* probe (Cy5), and 0.25 μM of universal sequence primer. Real-time PCR was performed on a Rotor-Gene Q real-time PCR system (QIAGEN, Hilden, Germany). Thermal cycling conditions were as follows: 95°C for 5 min and then 40 cycles of 95 °C for 15 s and 60 °C for 60 s. Heating and cooling rates were 20 °C per second and 15 °C per second, respectively. For each run, bisulfite-converted methylated (HCT116) and unmethylated genomic DNA were used as methylation controls. Non-template control was also included. Cycle threshold (C_T_) value was calculated using Rotor Gene Q software.

The cutoff value of C_T_ in real-time PCR assay for *SDC2* methylation was chosen based on the receiver operating characteristics curve (ROC) analysis on assay results. An optimal cutoff of C_T_ value for optimal sensitivity and specificity discriminating CRC patients from healthy subjects was determined at 40. Therefore, in interpreting LTE-qMSP assay for *SDC2* methylation, if C_T_ value was within 40 cycles, a sample was positive and treated as negative if the C_T_ value for *SDC2* methylation was undetectable. In analytic performance test for *COL2A1* with serially diluted genomic DNA (HCT116), LoD was 10 pg (~ 3 genome copies) at 36 of C_T_ value (data not shown). Thus, the test result was accepted only when the C_T_ value of *COL2A1* was ≤ 36.

### Statistical analysis

All statistical analysis was performed using MedCalc version 9.3.2.0 (MedCalc software, Ostend, Belgium). A *P* value of less than 0.05 was considered statistically significant. ROC, area under ROC (AUC), and 95% confidence intervals (CI) were calculated. Kruskal-Wallis test was performed to compare methylation levels and clinicopathological features.

## Results

### *SDC2* methylation status in various stages of colorectal tissues

**Fig. 1:**
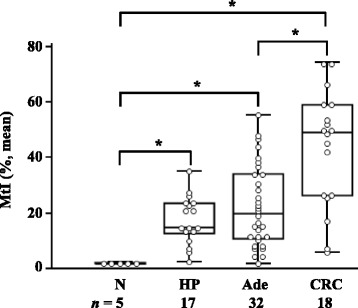
Assessment of methylation levels of the *SDC2* gene in colorectal tissues by bisulfite-pyrosequencing. Methylation level of the *SDC2* gene was evaluated in normal mucosa (N), hyperplastic (HP) and adenomatous polyps (Ade), and CRC tissues. The MtIs of each sample are represented by box and whisker plots. The difference in MtI of *SDC2* is statistically significant at *P < 0.01 calculated by Kruskal-Wallis test in adenomatous polyps vs. normal controls, hyperplastic polyps vs. normal controls, CRC vs. normal controls, and CRC vs. adenomatous polyps

Previously, we have reported that CpG sites of *SDC2* regulatory region are most differentially methylated between CRC and normal tissues [20]. To know how early such aberrant *SDC2* methylation might occur during tumorigenesis of CRC, *SDC2* methylation status was analyzed using bisulfite-pyrosequencing assay in tissue lesions with various severity, including hyperplastic polyps (*n* = 17), precancerous adenomatous (*n* = 32), and colorectal tumors (*n* = 18). In addition, normal mucosal tissues (*n* = 5) were included for comparison. When determining positive call at a cutoff value of 5.0%, a detection limit in pyrosequencing [23], frequency of aberrant *SDC2* methylation in cancerous tissues and precancerous lesions was significantly different from that of normal mucosa (*P* < 0.01). Positive *SDC2* methylation was observed in 100% (18/18) of primary tumors, 90.6% (29/32) of adenomas, 94.1% (16/17) of hyperplastic polyps, and 0% (0/5) in normal tissues. The level (MtI) of *SDC2* methylation was associated with increasing severity of lesions (Fig. 1). Overall mean MtI values (± SD) of normal mucosa, hyperplastic polyps, adenomatous polyps, and primary tumors were estimated at 2.0 ± 0.2, 17.6 ± 8.6, 22.4 ± 14.6, and 43.5 ± 21.0%, respectively (*P* < 0.01). These results indicated that *SDC2* methylation level was increased in accordance with severity of lesions.

### Development of me*SDC2* LTE-qMSP assay

Analyzing methylation status of the region of interest of target DNA, *SDC2* here, in stool DNA is a formidable challenge because target DNA exists at considerably low copy number with extremely high level of background noise from unrelated genomic DNA (derived from heterogeneous origins).

**Fig. 2:**
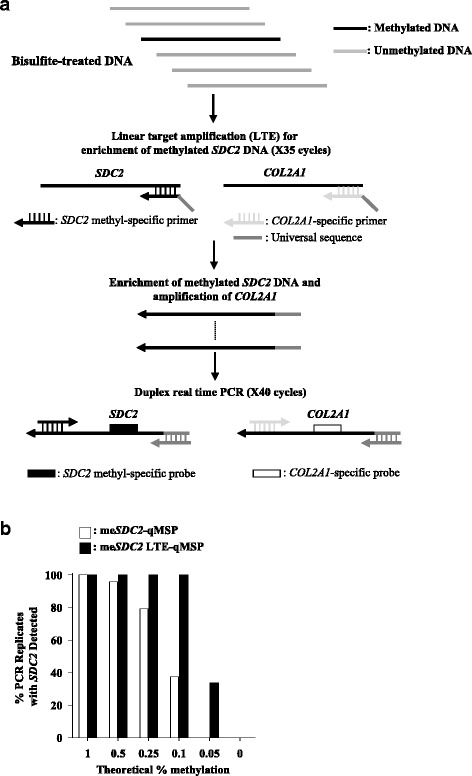
Schematic diagram and analytic performance of me*SDC2* LTE-qMSP. **a** Outline of me*SDC2* LTE-qMSP. LTE was first performed using two primers: *SDC2* and *COL2A1* antisense primers attached to a universal primer to enrich methylated *SDC2* DNA, and consecutive duplex real-time PCR was carried out using methylation-specific *SDC2* sense primer, *COL2A1*-specific sense primer, universal primer, and probes. **b** The LoD of me*SDC2* LTE-qMSP was compared with that of me*SDC2*-qMSP. Different amounts of HCT116 genomic DNA were diluted in unmethylated genomic DNA in total 20 ng of genomic DNA

To address this issue, we developed and launched this study to establish a new method demonstrating highly sensitive and specific quantitative detection of *SDC2* methylation in stool DNA, named as me*SDC2* LTE-qMSP covering CpG targets (+ 377 to + 500 bp), outlined in Fig. 2a.

This method implements two rounds of consecutive PCR consisting of (1) unidirectional linear amplification for target DNA and control by PCR and (2) quantitative real-time PCR for methylation analysis for target region of *SDC2*.

For the first round PCR, two primers, antisense-methylated *SDC2* specific and control *COL2A1* specific primers with the same universal tag sequence at 5′ end for second round PCR were simultaneously used for linear target enrichment of *SDC2* methylation and control *COL2A1.* In the second round of PCR, conventional qMSP by real-time PCR procedure for *SDC2* methylation was conducted using sense *SDC2* specific primer and universal sequence-matched primer with methylation-specific dual labeled probe. This method was selected to optimally enhance sensitivity and diminish non-specific amplification during intensive DNA amplification and compensate for frequent artifacts seen in two round PCR. To evaluate analytic performance of new method, me*SDC2* LTE-qMSP assays were conducted repeatedly (24 times) using bisulfite-treated mixture of genomic DNA (total 20 ng, ~ 6200 genome copies) as an initial template (different ratio of genomic DNA mixture), resulting in methylation percentages of 1.0, 0.5, 0.25, 0.1, 0.05, and 0%, respectively (between fully methylated and unmethylated genomic DNA).

Regarding specificity testing, various amounts (10^5^ to 10^9^ copies) of plasmids (representing fully unmethylated *SDC2* DNA sequence) were implemented as a template for the assay. The me*SDC2* LTE-qMSP detected *SDC2* methylation levels as low as 0.05% (~ 3 genome copies) and revealed no cross reactivity, even with excess 10^9^ copies of plasmids (data not shown). If LoD is defined as “a condition showing positive in more than 95% of 24 repeated assays” [26], LoD of me*SDC2*-qMSP was 0.1% methylation corresponding to 20 pg (~ 6 genome copies in ~ 6200 copies of total template) in which detection rate was 100% (Fig. 2b).

To compare the performance of LTE-qMSP with conventional qMSP, qMSP assays (me*SDC2*-qMSP) for *SDC2* methylation covering CpG targets (+ 377 to + 498 bp) [20] was evaluated in the same way with different ratio of genomic DNA mixtures, resulting in that LoD of me*SDC2*-qMSP was 0.5% methylation equivalent to 100 pg (~ 30 genome copies). This data indicated that LTE-qMSP assay achieved 5-fold improvement in LoD compared with qMSP for *SDC2* methylation. As well, C_T_ value for methylated *SDC2* was detected several cycles earlier by the me*SDC2* LTE-qMSP method, when compared to that of the me*SDC2*-qMSP assay for all dilutions of methylated *SDC2* (Table 3). Linear regression analysis of me*SDC2* LTE-qMSP for *SDC2* methylation demonstrated reproducible linearity, with a correlation coefficient of *R* ^2^ = 0.992 (data not shown).

**Table 3:** Comparison of analytic performance between a me*SDC2* LTE-qMSP and me*SDC2*-qMSP

DNA Concentration (pg)	me*SDC2* LTE-qMSP detected	me*SDC2* LTE-qMSP Avg C_T_	me*SDC2*-qMSP detected	me*SDC2*-qMSP Avg C_T_
200	24 out of 24	25.0	24 out of 24	32.8
100	24 out of 24	26.0	23 out of 24	33.2
50	24 out of 24	27.3	19 out of 24	34.9
20	24 out of 24	29.6	9 out of 24	35.9
10	8 out of 24	33.9	0 out of 24	N.D
Negative control	0 out of 24	N.D	0 out of 24	N.D

*N.D* not detected

### Feasibility test of using *SDC2* methylation in stool DNA-based assay for detection of CRC and precancerous lesions

**Fig. 3:**
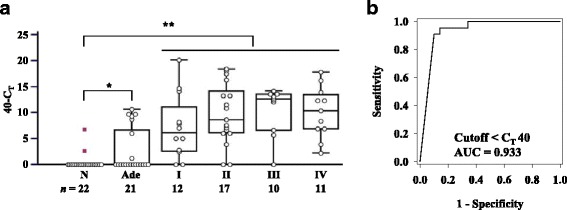
Methylation status of *SDC2* in stool DNA by me*SDC2* LTE-qMSP test. **a** me*SDC2* LTE-qMSP test was performed in stool DNA from CRC patients in varying stages, adenoma patients (Ade), and healthy normal subjects (N). Distribution of the relative level of *SDC2* methylation was expressed in C_T_ values as 40-C_T_ for each sample. A higher 40-C_T_ represents a higher methylation level of *SDC2.* It is represented as 0, if the *SDC2* C_T_ was not detected. Methylation status of the *SDC2* gene is plotted as box and whisker plots. The difference in methylation level of *SDC2* was statistically significant at **P < 0.01 and *P < 0.05 calculated by Kruskal-Wallis test in CRC patients vs. healthy normal subjects and adenoma patients vs. healthy normal subjects, respectively. **b** ROC curve was plotted for CRC patients vs. healthy normal subjects. The cutoff value for methylation-positive and AUC are indicated in the plot

To determine whether measurement of *SDC2* methylation in stool DNA was capable of detecting CRC and adenoma, me*SDC2* LTE-qMSP was performed using stool DNA from 50 CRC patients at various stages and 21 patients with small size (< 1.0 cm) of adenoma. In addition, stool DNA from 22 healthy normal subjects were included. me*SDC2* LTE-qMSP showed significant higher frequency of aberrant *SDC2* methylation in stool DNA from both CRC (*P* < 0.01) and adenoma (*P* < 0.05) patients compared to that in healthy normal subjects (Fig. 3a). Meanwhile, 20 out of 22 healthy subjects showed an absence of methylation, indicating that *SDC2* methylation in stool samples had high specificity. To evaluate clinical performance of *SDC2* methylation in detection of CRC, ROC curve was constructed by optimizing sensitivity and specificity using assay results. ROC analysis determined optimal cutoff value of C_T_ at 40 with AUC of 0.933 (95% CI 0.848–0.978) (Fig. 3b). Overall sensitivity of testing for detection of CRC was estimated at 90.0% (45/50, 95% CI 78.2–96.6%) with specificity of 90.9% (2/22, 95% CI 70.8–98.6%). Sensitivities for individual stages I, II, III, and IV were 83.3% (10/12), 88.2% (15/17), 90.0% (9/10), and 100% (11/11), respectively. *SDC2* methylation was also detected in 7 (33.3%) of 21 adenomas < 1.0 cm. Thus, the sensitivity tended to be gradually increased in accordance with the severity of neoplasms.

## Discussion

The results of this study indicate that quantifying the methylation level of *SDC2* in exfoliated epithelial cell-derived DNA isolated from human stool would be a new useful noninvasive screening tool for early-stage CRC.

Screening tests have effectively reduced disease related CRC mortality, and there are currently several options available to screen the CRC. Although immunochemical FOBT is the most widely used method to detect early-stage CRC, it is limited by its low sensitivity of 65.8% [6]. A colonoscopy offers the most complete and sensitive test that is currently available. However, a colonoscopy is an invasive method to screen CRC that requires extensive bowel preparation, and it could lead to serious complications, so many patients find it undesirable. Thus, stool DNA-based molecular marker tests have been recently proposed as a new alternative to screen early-stage CRC [27].

Aberrant DNA methylation of some genes has been known to occur early during tumorigenesis. Therefore, specific methylation sites have been considered as potential biomarkers for the early diagnosis of cancer [28]. Previously, we identified that the CpG island of *SDC2* that is normally unmethylated was one of most predominantly methylated DNA sites in tumors of CRC patients, regardless of stage. The 5′ regulatory region of *SDC2* plays a role as diagnostic biomarker for early detection of CRC because this region was frequently hypermethylated in CRC, while unmethylated in healthy normal controls. We then demonstrated that aberrant methylation status of *SDC2* can be readily detected in DNA derived from the bodily fluids, such as serum and stool, of patients with CRC [20, 29], indicating its potential as a noninvasive molecular diagnostic biomarker for the early detection of CRC. A frequent *SDC2* hypermethylation was in tissues of gastric cancer, and aberrant methylation of *SDC2* was correlated with diffuse-type and mixed-type gastric cancers [30]. *SDC2* hypermethylation was in HPV-positive primary tumor of head and neck squamous cell cancer and glioma multiforme [31, 32]; however, details of clinical performance of *SDC2* methylation test were not addressed in these studies.

In the present study, we attempted to determine whether stool DNA-based test using *SDC2* methylation as a biomarker was a viable option to detect CRC. We first confirmed that the abnormal methylation of *SDC2* occurs in almost all CRC tissues regardless of stage and is observed also in biopsies of various precancerous lesions while not detected in normal mucosal tissues. The methylation level of *SDC2* in tissue samples tend to increased according to the severity of lesions.

Developing sensitive stool DNA-based methylated DNA tests for early detection of CRC is challenging for a number of reasons. Normally, stool contains a mixture of cells such as exfoliated epithelial cells from colon mucosa and others and in case of CRC patients a small fraction of the neoplastic cells shed from tumor lesions [33–35]. Furthermore, DNA derived from bacteria and diets tend to increase heterogeneity of stool-derived DNA [36–38]. To complicate matters, the presence of various interference/contaminating factors in the stool might inhibit PCR reactivity. Keeping in mind these various issues, we here introduced a LTE-qMSP, a highly sensitive and specific methylation detection method that employs two-step PCR procedures on bisulfite-treated purified stool DNA and combines linear target enrichment for methylated *SDC2* and target amplification via real-time PCR for *SDC2* methylation. Pyrosequencing measures methylation status of individual and multiple CpG sites. LoD of pyrosequencing is as low as 5% fraction of methylated DNA in the background of unmethylated DNA [23]. Our new LTE-qMSP measures absolute methylation level (C_T_) of multiple CpG sites covered by methylation-specific primers and probe. LTE-qMSP has a LoD of 0.1% fraction of methylated DNA in the background of unmethylated DNA. In our preliminary study, pyrosequencing revealed low sensitivity for detection of *SDC2* methylation in stool DNA (data not shown). Thus, LTE-qMSP had an advantage of higher detection sensitivity for *SDC2* methylation compared with pyrosequencing.

In this study, we applied LTE-qMSP assay to measure *SDC2* methylation status in stool DNA from 50 CRCs, 21 adenomas, and 22 healthy individuals. A single reaction of me*SDC2* LTE-qMSP revealed overall sensitivity of 90.0% for detection of CRC with slightly lower sensitivity for early stage than later stage CRCs with 83.3% sensitivity for stage I and a specificity of 90.9% for healthy subjects. In terms of specificity matter, 9.1% of healthy individuals revealing *SDC2* methylation-positive are counted as false-positive. However, considering that not all healthy subjects were verified with colonoscopy as adenomatous polyp-free and more than 30% of asymptomatic adults older than 50 had polyps [39], possibility that those healthy subjects revealing *SDC2* methylation-positive have polyp(s) cannot be excluded. Specificity should be further evaluated in a number of colonoscopy-determined normal controls in future studies. In addition, we examined frequency of aberrant *SDC2* methylation in stool DNA from patients with a small size of adenomas (< 1.0 cm) and estimated 33.3% of sensitivity. Notably, as compared to a single test when another reaction of me*SDC2* LTE-qMSP was applied on the remaining stool DNA and the sample classified positive based on at least one positive for methylation out of the two reactions, the sensitivity increased from 33.3 to 42.9%, as compared to a single test (data not shown). This suggests that multiple tests of LTE-qMSP for *SDC2* methylation in stool DNA probably increase clinical sensitivity in detecting early stage of CRC as well as advanced polyps. Since the ultimate aim of screening is to prevent of CRC, screening test should be effective to detect those precancerous lesions at the risk of CRC progression [17]. When small size (< 1.0 cm) of adenomas was detected, the patients will be generally subjected to observation. However, large adenomas > 1.0 cm or multiple adenomas are more likely to progress to CRC, and they are currently subjected to be removed during colonoscopy. Therefore, it will be worthy to investigate how well *SDC2* methylation test can detect advanced or multiple precancerous lesions in future studies.

Several groups have reported studies examining DNA methylation biomarker performance in stool DNA for early detection of CRC or precancerous lesions [9, 16, 40, 41]. For example, *SFRP2* has shown a sensitivity of 77–90% with specificity of 77% to detect CRC in stool samples, indicating an excellent sensitivity but unsatisfactory specificity [40]. *HIC1* and *vimentin* genes have sensitivities of 42 and 46%, respectively, with specificities of 100 and 90%, respectively, to detect CRC in stool DNA [16, 41]. Huang et al. [4] analyzed the methylation of multiple genes (*SFRP2*, *HPP1*, and *MGMT*) in stool DNA from a large population. The sensitivity and specificity to detect CRC in the three combined genes was 96.2 and 95.8%, respectively.

On the other hand, Ahlquist et al. [17] reported a next generation stool DNA test using multiple markers, including four methylated genes (*vimentin*, *NDRG4*, *BMP3*, *TFPI2*) and *KRAS* mutations. This test was able to detect CRC and precancerous adenoma with sensitivities of 85 and 54%, respectively, at a specificity of 90% [17]. In addition, a recent study by Imperiale et al. [19] reported a stool-based multi-target test composed of seven sites of *KRAS* mutation, two methylation markers (*NDRG4* and *BMP3*), and FIT. Multi-target stool testing showed a sensitivity of 92.0% to detect CRC and 42.4% for advanced precancerous lesions at a specificity of 87.0%.

Mitchell et al. [42] recently performed genome-wide methylation analysis with DNA microarray in CRC tumors compared to matched-normal tissues and observed that *SDC2* gene was frequently methylated tumors. Subsequently they confirmed that *SDC2* methylation was evident in 80% or more of the tested cancer tissues by qMSP. Very recently while we were preparing this manuscript for publication, Niu et al. [18] published a paper showing that methylation tests of *SDC2* in fecal DNA detects CRC and advanced adenoma (≥ 1.0 cm) with sensitivities of 81.1 and 58.2%, respectively, at a specificity of 93.3%. Both data are comparable to ours and indicating that *SDC2* methylation event has a high potential as an early diagnostic biomarker for CRC.

In a tissue level, *SDC2* methylation events have been observed in all tumor tissues of most CRC patients but have never been observed in normal colon tissues by pyrosequencing-based methylation assay having a detection limitation of 5% [23]. In the stool DNA test, ROC analysis on assay results determined an optimal cutoff for *SDC2* methylation for detecting CRC at C_T_ value of 40. Thus, a sample was treated as positive if C_T_ value for *SDC2* methylation was within 40 and considered as negative if the C_T_ value for *SDC2* was not detectable. Healthy individuals showed a very rare frequency (9.1%) of methylation-positive. Therefore, *SDC2* methylation can be easily added to the other biomarkers reported above, thereby improves clinical performance for the early diagnosis of CRC, without losing specificity.

This study has a limitation in the small size of the patient and normal groups. Therefore, future large-scale of investigation for clinical validation of *SDC2* methylation and intensive evaluation of the ethnic or regional difference will be warranted.

## Conclusions

We previously identified a novel potential methylation marker *SDC2* for the early detection of CRC. In this study, to validate *SDC2* methylation is able to detect patients with CRC and precancerous lesion using stool DNA, we developed a sensitive stool-based me*SDC2* LTE-qMSP. Our results demonstrate that abnormal *SDC2* methylation is a frequent event in precancerous adenomas and CRC but is negative in normal mucosa. Our results suggest that *SDC2* methylation is a new potential diagnostic biomarker for noninvasive screening of CRC.

## Abbreviations

CRC: Colorectal cancer
FIT: Fecal immunochemical test
FOBT: Fecal occult blood test
LoD: Limit of detection
LTE-qMSP: Linear Target Enrichment-quantitative methylation-specific PCR
MtI: Methylation index
*SDC2*: The protein encoded by this gene is a transmembrane (type I) heparan sulfate proteoglycan and is a member of the syndecan proteoglycan family. The syndecan-2 protein functions as an integral membrane protein and participates in cell proliferation, cell migration, and cell-matrix interactions via its receptor for extracellular matrix proteins
